# A cross-sectional study on the prevalence of HIV and hepatitis B virus co-infection among students of a tertiary institution in Ekiti State, Southwest Nigeria

**DOI:** 10.11604/pamj.2023.44.7.31416

**Published:** 2023-01-05

**Authors:** Olakunle Amos Ojerinde, Stephen Kayode Simpa Ojo, Uchenna Linda Udewena, Seye Julius Oladeji

**Affiliations:** 1 University Medical Centre, Federal University Oye-Ekiti, Ekiti State, Nigeria,; 2 Drug Discovery and Infectious Diseases Research Group, Department of Microbiology, Federal University Oye-Ekiti, Ekiti State, Nigeria

**Keywords:** Prevalence, hepatitis B virus, HIV, infectious diseases, IEC- information, education, technology

## Abstract

Nigeria aims to eradicate public-health threats such as HIV/AIDS and hepatitis B virus (HBV) by 2030. However, to achieve the short- and medium-term response target, and end the epidemic by 2030, there is the need to monitor and estimate the population level of HIV and HBV epidemic trends to boost the country's strategic framework's chances of success. Hence, we uncovered the prevalence of HIV and HBV among full-time, newly admitted undergraduate university students in Southwestern Nigeria between 2015 and 2017. In this regard, 4 ml of blood samples was collected from each subject into Ethylene Diamine Tetraacetic Acid (EDTA) bottles and were allowed to stand for one hour. Samples were allowed to separate into plasma and corpuscles on the bench. HIV screening was done using an immunochromatographic method via a highly sensitive kit DETERMINE® (Abbott Diagnostic Division, Netherlands) and were later confirmed using Enzyme Linked Immunosorbent Assay (ELISA) Uni-Gold® manufactured by Trinity Biotech Plc, Ireland. HBV screening was carried out using an immunoassay method for the detection of the hepatitis B surface antigen (HBsAg). Out of the 4,623 subjects recruited, 2,545 were male while 2,078 were female. The overall prevalence of HIV was found to be 0.13% while that of HBV was 2.23%. Conclusively, although HIV was found to be less prevalent among the study as compared to HBV; however, the higher transmission propensity of HBV necessitates even more urgent efforts to eradicate the infectious diseases.

## Introduction

In the context of HIV/AIDS, educational institutions are supposed to impart to students the knowledge, abilities, and attitudes that encourage safe behaviours so that they can prevent HIV infection [1]. Nigeria is one of the African countries with no formal HIV/AIDS policies in place at tertiary institutions [2]. However, it is crucial to emphasize that efforts to eradicate AIDS require higher institutions to carry out both social and moral duties. Hence, in order to establish control over the threat, creating an institutional policy for addressing AIDS is a step in the right direction [2]. According to the 2017 revision of the World Population Prospects, the total Nigerian population was estimated at 185,989,640 in 2016, with adolescents and young adults (aged 15-39 years) accounting for the majority of this population. More importantly, these people are more socially active, which makes them more vulnerable to infectious diseases like HIV and HBV [3].

Unfortunately, the number of HIV tests and counselling sessions provided in Nigeria has remained below the advised level over time. In light of this, women aged 15 to 49 are more than twice as likely to be living with HIV than males (1.9% vs 0.9%), despite Nigeria's overall HIV prevalence among people aged 15 to 49 being 1.4%. The disparity in the prevalence of HIV in women and men is largest among younger persons, with young women aged 20 to 24 years more than three times as likely to be infected as young males in the same age range. According to the most recent rankings, Nigeria is one of the nations with the most unequal power distributions in the world, coming in at number 122 out of 144 nations for the size of its “gender gap”. In the context of gender power disparities, women commonly experience challenges choosing their own sexual partners, using contraception, deciding how many children to have, and managing their own healthcare, all of which increase their risk of contracting HIV [3].

While it has been shown that blood transfusion and heterosexual transmission account for around 80% and 5% of HIV infections, respectively, mother-to-child transmission, needle sharing, and scarification, to mention a few, account for the remaining 15% [4]. The behavioral attitude of Nigerians in regard to sex is the main cause of the epidemic. Additionally, high levels of poverty, harmful traditional practices, high stigma and patient discrimination, low levels of education, high levels of sexually transmitted infections, mother-to-child transmission, widespread high-risk sex, low risk perception, high rates of sexually transmitted infections (STIs), wife hospitality, spouse sharing, and polygamy are some of the observed factors causing the pandemic to spread [5].

Despite the introduction of universal HBV vaccination and effective antiviral therapy, the estimated overall sero-prevalence of HBV surface antigen remains high in Nigeria at 8.1%. HBV is very similar to HIV in the ways it is transmitted: through direct blood to blood contact and through sexual activity. However, blood levels of HBV are much higher than for HIV, making the virus much easier to transmit in certain situations. Additionally, despite the fact that HBV infection is widespread in Nigeria, little is known about the virus's epidemiology among young people and student populations in spite of the importance of this information for creating successful intervention programs. Before any clinical signs appear, a person may have HBV infection for 30 years or more. Sero-conversion from HBeAg to HBeAb and serum elimination of HBV deoxyribonucleic acid (DNA) are linked to illness remission [6].

To this end, while efforts are currently being made in Nigeria to eradicate HIV and HBV infections, it is undoubtedly vital to track and estimate the population level of HIV and HBV epidemic trends in order to boost the country's strategic framework's chances of success. As a result, this study uncovered the prevalence of HIV and HBV among the newly admitted students at a tertiary university in southwestern Nigeria, taking into account the significant role played by the university students in the spread of these infectious diseases across the country.

## Methods

**Study population:** a total of 4,623 samples were collected from newly admitted students of the Federal University Oye-Ekiti, Ekiti State, South-West Nigeria over a period of about 3 years (2015-2017). Of the 4,623 students, 2,268 were females and 2,355 were males of age range of 15-39 years.

**Inclusion/exclusion criteria:** these include being a full-time and newly admitted undergraduate student at the Federal University Oye-Ekiti State and willingness to volunteer a blood sample.

**Study design:** this cross-sectional survey included only first-year male and female students at Federal University Oye-Ekiti. After obtaining the participants' consent, blood samples were taken from willing students for testing. The collecting point was the university health center.

**Sample collection and preparation:** venepuncture method was used to collect 4 ml of whole blood from the participating undergraduates into 1.5 mg/mL EDTA (Ethylene Diamine Tetraacetic Acid). Thereafter, the samples were allowed to separate into plasma and corpuscles.

### Sample testing

**Human immunodeficiency virus (HIV) screening; baseline testing:** in accordance with the serial algorithm adopted for the study and recommended by Amechi *et al*. [7], all samples were initially screened for the presence of HIV types 1 and 2 antibodies using an immunochromatographic method via the DETERMINE® (Abbott Diagnostic Division, Netherlands) highly sensitive rapid test kit, as described by Daramola *et al*. [8] and Amechi *et al*. [7]. After removing the test pouch's protective foil cover, 50 L of plasma was poured onto the sample-pad. The result was read in accordance with the manufacturer's instructions after allowing enough time for the plasma to go through the control and test windows. ELISA Immunosorbent Assay (EIA): The EIA method, which was able to identify and confirm the presence of HIV antibodies, was used to confirm the true positivity of all the participants who tested positive at baseline screening. This was accomplished utilizing Uni-Gold®, a product of Ireland's Trinity Biotech Plc.

**Hepatitis B surface antigen test:** hepatitis B surface antigen was detected by using an immunoassay technique (HBsAg). After removing the test pouch's protective foil cover, 50 L of plasma was poured onto the sample-pad. The result was read in accordance with the manufacturer's instructions after allowing enough time for the plasma to go through the control and test windows.

**Statistical analysis:** this was carried out using SPSS version 16, and the results or outcomes were shown in figures, tables, and graphs. The prevalence of HBsAg and HIV viral infections were determined from the proportion of seropositive individuals in the population under consideration and expressed as a percentage.

## Results

**Figure 1:**
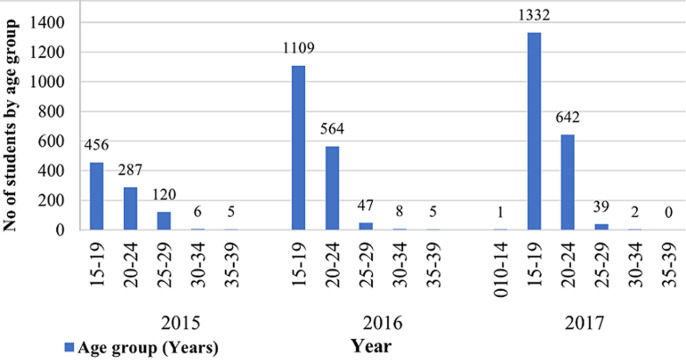
distribution of total subjects by age brackets

**Figure 2:**
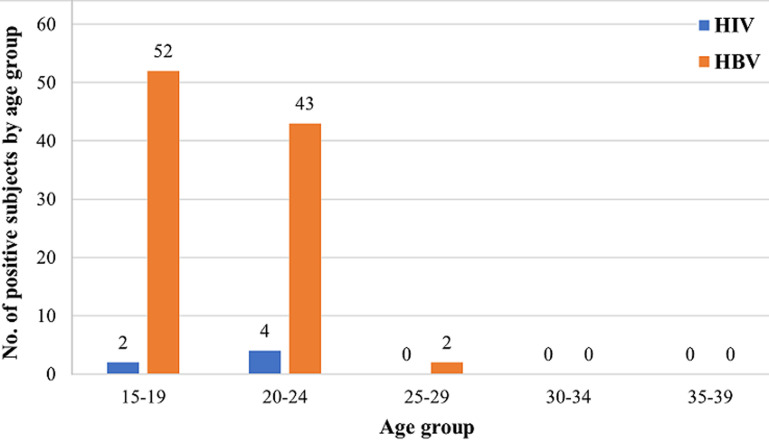
distribution of HIV and HBV positive subjects by age group

**Figure 3:**
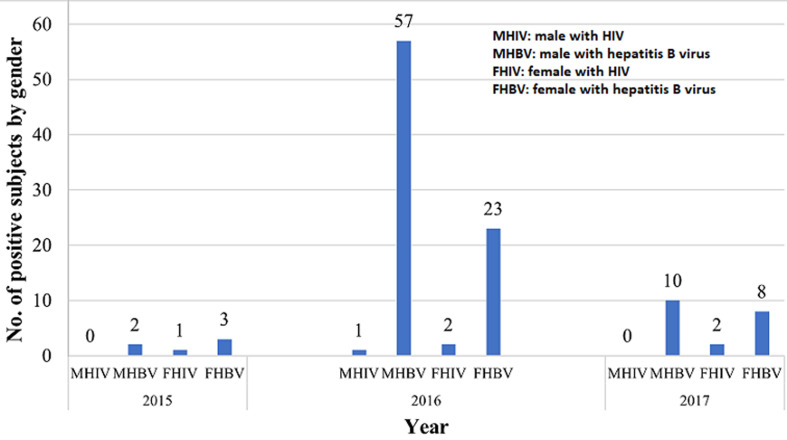
distribution of HIV and HBV positive subjects by gender

The subjects comprised of a total of 4,623 participants having 2,545 (55.05%) males and 2,078 (44.95%) females (Table 1). A total of 1 (0.02%) subject was within 10-14 years; 2,897 (62.67%) subjects were within 15-19 years; 1,493 (32.30%) were within 20-24 years, 206 (4.46%) were within 25-29 years while 16 (0.35%) and 10 (0.22%) were within the 30-34 and 35-39 years, respectively (Figure 1). Of the 6 HIV positive participants, 2 (33.33%) were within the age range of 15-19 years while 4 (66.67%) were within the age range of 20-24 years. The age range distribution of HBV positive subjects within 15-19 years were 52 (53.61%), 43 (44.33%) were within 20-24 years, and 2 (2.06%) within 25-29 years (Figure 2). Only 1 female subject was HIV positive in 2015; 2 females and 1 male in 2016, and 2 females in 2017. However, among the HBV positive subjects, 2 males and 3 females were tested positive in 2015, 57 males and 23 females in 2016, and 10 males and 8 females in 2017. This study revealed high prevalence of HBV among student population especially with male subjects as compared to HIV. Total HIV prevalence from the subjects was 0.13% while total HBV prevalence was 2.23% (Figure 3).

**Table 1:** distribution of total subjects by gender

Year	Male (%)	Female (%)	Total (%)
2015	532 (60.87)	342 (39.13)	874 (100)
2016	1030 (59.43)	703 (40.57)	1733 (100)
2017	983 (48.76)	1033 (51.24)	2016 (100)
Total	2545 (55.05)	2078 (44.95)	4623 (100)

## Discussion

Studies have shown that most university students do not know their HIV and HBV status, and this increase the chances of infection and spread. Hepatitis B virus and HIV share a common risk factor and common mode of transmission, so it is rationale to study the prevalence of these two together. Reports from Isa *et al*. [9] (9.2%), Alo *et al*. [10] (15.5%), Tula and Iyoba [11] (31.5%), Pennap *et al*. [12] (11.5%), Olalekan [13] (6.2%) suggests that HBV is endemic in Nigeria and also within Nigerian tertiary institutions. In this present study, results showed a higher prevalence of HBV (4.62%) for the year 2016 compared to the year 2015 (0.57%) and year 2017 (0.89%). The total HBV prevalence for the 3 years is (2.23%) which means that the study population has intermediate endemicity (2%-7%) of HBV.

This result correlates with that of Akinbolaji *et al*. [14], Simidele *et al*. [15], Mboto and Edet [16], Enitan *et al*. [17] and Olalekan [13], who also reported moderate endemicity of 6.16%, 4.5%, 4.7%, 1.5% and 6.2%, respectively and much lesser than the reports of Aminu *et al*. [18], Musa *et al*. [19], Olayinka *et al*. [20], Pido *et al*. [21], Mohammed *et al*. [22], Ndako *et al*. [23], who reported high endemicity of 12.5%, 13.6%, 12.2%, 11.0%, 9.7%, 18.4%, respectively. The intermediate (2.23%) prevalence of HBsAg obtained in this study suggests that hepatitis B is somewhat common among students in the campus. Hepatitis B virus can be transmitted via contact with all bodily fluid (including saliva, semen, sweat, breast milk, tears, urine, vaginal secretions, and faeces), and by frequent and prolonged close personal contact with an infected person [18].

This study also showed that HBV is more common in males than females, which correlates with the reports of Akinbolaji *et al*. [14], Aminu *et al*. [18], Lado *et al*. [24], Enitan *et al*. [17], and Simidele *et al*. [15] but contrasts with the findings of Mboto and Edet [16], and Ndako *et al*. [25]. This could be due to disparity in sample size, sample population and the varying levels of engagement in the risk predisposing practices across populations and communities. Uneke *et al*. [26] suggested that both sexes are equally susceptible to HBV infection while gender may not necessarily be an important epidemiological determinant of HBV infection. Conversely, in the study conducted by Mehmet *et al*. [27], higher prevalence was observed among male subjects as compared to female subjects in both rural and urban areas. The authors also noted that male sex was an important risk factor for HBsAg positivity, which is consistent with the findings of the present study. Additionally, the reason for the high HBV infection rate among the males may be due to risky habits such as multiple sexual partnership and poor educational background which affect enlightenment and promote the transmission of the virus. Educational background may assist at prevention of transmission by enlightenment strategies [25].

Furthermore, the highest prevalence was found within the age group 15-19 years followed by 20-24 years, which suggests that most of these subjects may have acquired the infection though risky lifestyles or behaviours. Testing and treatment of existing infection does a substantial impact as vaccinating older age group has negligible sustained effort in curbing increasing HBV infection as reported by McNaughton *et al*. [28]. Nigeria is one of the African countries with no formal HIV/AIDS policies in place at tertiary institutions [2]. Although there is information on the prevalence of HIV in various states and among different populations in Nigeria, there is however paucity of data on the prevalence among students of tertiary institutions. The HIV/AIDS situation in the university by the nature of their establishment, universities should take the threat posed by HIV/AIDS seriously without being prompted [29]. For a start, their principal clients are students, most of whom are in the 19-30 years age group. This is the age range within which HIV infection normally peak in most countries and so the need to limit its spread is of paramount importance. Generally, half of all those who become infected with HIV are young people under age 25.

In this study however, the prevalence of HIV infection among the university newly admitted students is quite low (0.13%) which is in line with the overall prevalence of less than 1% reported according to the National HIV/AIDS Indicator and Impact Survey [30] in the state. This very low prevalence of HIV may be because the study populations are freshers, which translates that most, or a good number of them have been staying under the watchful eyes of their parents or wards and so there is no such liberty to engage in high-risk behaviours that may expose them to the virus. The prevalence of HIV in this study conforms with that of Gayle *et al*. [31] (0.2%) but deviates a little bit from that reported by De Beer *et al*. [32] (1.8%) among students of Federal University in Namibia, and that of Mengistu [33] (2.5%). In addition, universities offer conditions ideal for the spread of HIV. Being a “captive” population, they contain a pool of HIV infected people [29]. Multiple sexual partnership is also likely on university campuses. Furthermore, partner mixing is another likely feature as students alternate between different sets of sexual partners during vacation and during academic sessions. The liberal atmosphere on university campuses is an added dimension to the whole episode. Some students may be enjoying independence from the watchful eyes of their parents for the first time.

This study has significant implications for achieving millennium development goals 4, 5, and 6 related to HIV in Nigeria and other sub-Saharan countries. There is a potential for an increased trend if adequate HIV services are not provided in institutions. Differences in sample populations between the studies may account for the variation in findings [9].

## Conclusion

Ensuring that youths are not infected is the greatest strategy for preventing the spread of HIV and HBV in tertiary institutions. The stigmatization associated with HIV/AIDS and the usual fear in going for the HIV test necessitates adequate advocacy and sensitization through the use of different IEC materials and media. Furthermore, it is necessary to include sex education among the first-year general studies courses to enlighten new students on issues on HIV/AIDS and other sexually transmitted diseases such as HBV and on the importance of HIV and HBV testing.

### What is known about this topic

- *Nigeria is one of the African countries with no formal HIV/AIDS and HBV policies in place at tertiary institutions;*
- *Youths, including university students orchestrate immense roles in the spread of HIV and HBV in Nigeria;*
- *In recent years, Nigeria has been pragmatic about eliminating HIV and HBV in the country with little or no information on the epidemic trends of the infectious diseases amid her citizens.*

### What this study adds

- *We uncovered the prevalence of HIV and HBV among full-time newly admitted undergraduate student over a period of about 3 years;*
- *This study has significant implications for achieving millennium development goals 4, 5, and 6 related to HIV and HBV in Nigeria and other sub-Saharan countries.*
